# Assessing Changes in Ecosystem Service Values in Response to Land Cover Dynamics in Jiangxi Province, China

**DOI:** 10.3390/ijerph17093018

**Published:** 2020-04-27

**Authors:** Xinmin Zhang, Hualin Xie, Jiaying Shi, Tiangui Lv, Caihua Zhou, Wangda Liu

**Affiliations:** 1 Institute of Ecological Civilization, Jiangxi University of Finance and Economics, Nanchang 330013, China; xiehualin@jxufe.edu.cn (H.X.); skying1997@163.com (J.S.); audrey0515@163.com (C.Z.); 2 School of Tourism and Urban Management, Jiangxi University of Finance and Economics, Nanchang 330013, China; lvtiangui@jxufe.edu.cn (T.L.); liuwangda966@163.com (W.L.)

**Keywords:** land cover dynamics, ecosystem service values, ecosystem service trade-off degree, Jiangxi province

## Abstract

This paper examines the ecosystem service values of Jiangxi province, China using the benefit transfer approach. The land cover dynamics results show that cropland and forest are the main land cover types in Jiangxi province. Urban land drastically increased after 2000, expanding from 846.54 km^2^ in 2000 to 2317.48 km^2^ in 2015. Forest and water obviously decreased across the study periods. Consequently, the total ecosystem service values decreased from 37.91 × 10^10^ Yuan in 1995 to 35.27 × 10^10^ Yuan in 2015. The values showed a declining trend, especially during the 1995–2000 period. The largest declines in ecosystem service values were caused by decreases in forest and water cover. Regulating services experienced the largest declines in ecosystem services value. Moreover, water supply showed the largest decline in ecosystem service value between 1995 and 2015. Not surprisingly, food production increased in the whole period, especially in the 1995–2000 period. Forest and cropland played the most important roles in the total ecosystem service values of Jiangxi province. We then discussed the relationship among ecosystem services based on the ecosystem service trade-off degree. The results show that the dominant relationship among ecosystem services in Jiangxi province was synergy; thus synergy mostly occurred in all ecosystem services except for food production from 1995 to 2015. However, during the 1995–2000 period, trade-offs mainly existed in both food production and waste treatment. The proportion of synergy greatly increased in the 2000–2015 period, and the synergistic relationship between waste treatment and other ecosystem services increased. However, the trade-off relationship between food production and other ecosystem services still has not improved, which should be concerned in the future. Changes in the percentage share of cropland showed a declining trend; thus, the potential risk of cropland loss should be monitored.

## 1. Introduction

Ecosystem services include ecosystem products (e.g., food) and services (e.g., waste assimilation), and the term refers to the benefits directly or indirectly obtained from ecosystem functions [1]. By providing natural environmental conditions and utility for human survival [2], ecosystem services are closely related to human well-being. Land is the basic carrier of socioeconomic activities. Land use/cover change affects ecosystem service functions by changing biodiversity, ecosystem structure, and habitat, and land use/cover change is one of the most important ways in which human activities affect ecosystems [3]. In 2005, the Millennium Ecosystem Assessment (MEA) report noted that in the past 50 years, the rapid growth of human demand for nature has led to the degradation and loss of ecosystem services, which may further deteriorate and ultimately endanger the long-term development of human beings [4]. To better coordinate the relationship between human activities and the ecological environment, it is valuable to evaluate ecosystem services from the perspective of economic value and measure the impact of land cover dynamics on ecosystem service values.

Ecosystem services have been a hot topic in environmental studies [5,6]. With rapid economic development and the continuous improvement of people’s living standards, human activities result in the deterioration of ecosystem services. Subsequently, a variety of environmental protection policies have been implemented to guarantee and improve regional ecological services in the context of rapid new urbanization [7,8]. In China, the value of ecosystem services per unit area shows obvious spatial characteristics: high in the south and low in the north; high in the east and low in the west; and gradually increasing from the northwest to southeast [9]. The spatial variations can be explained by the fact that the climate is dry and the vegetation is sparse in Northwest China; the recent rapid industrialization and urbanization in Central and East China has increased the area of artificial surface and reduced the natural vegetation [10,11]. Furthermore, with the implementation of ecological restoration projects (e.g., Grain for Green Project), regional ecosystem services have been improved [8].

In recent years, the trade-off and synergistic relationships among ecosystem services have become an important topic in the realm of ecosystem services research [12,13]. When one service responds negatively to a change in another service, this relationship is called a trade-off [4]; when both services change positively in the same direction, this relationship is called a synergy [14]. The ecosystem service trade-off degree has been used in certain studies to evaluate the trade-off and synergistic relationships [15,16], and it is an approach based on the linear fitting of data to reflect the direction and degree of interaction among ecosystem services [15,17]. Thus, the examination of ecosystem service trade-off degree can provide a valuable direction for future consideration of ecosystem services.

Several studies have shown that ecosystem service values are generally estimated based on the benefit transfer method through land use/cover data [18,19,20]. The land cover dataset of the European Space Agency’s Climate Change Initiative (ESA CCI) is widely used to study landscape changes at different scales [21,22,23,24]. However, the land cover change information provided by the ESA CCI land cover dataset is rarely used for ecosystem services. Currently, a global ecosystem service study was estimated based on ESA CCI land cover products [25]. It is worth mentioning that the ESA CCI land cover products provide 24 years of annual data (i.e., from 1992 to 2015). Therefore, ESA CCI land cover products are more suitable for the study of long time series.

Jiangxi province is an important ecological security barrier in southern China. Since 2001, Jiangxi province has put forward the development strategy of accelerating industrialization and urbanization, and the implementation of national strategies, e.g., the Poyang Lake Eco-Economic Zone in 2009 and the revitalization of the Gannan Soviet Area in 2012. The speed of economic growth in Jiangxi province greatly increased. However, the conflict between the ecological environment and economic growth is increasingly prominent. Some projects performed in Jiangxi province and have attached great importance to the protection of the ecological environment. Jiangxi province started the Grain for Green Project in 2001, and 11.35 million mu of the first round of tasks had been completed by the end of 2014, further improving forest coverage and maintaining land ecological security. The ecological protection red line was officially issued in July 2018, with an area of 46,876 square kilometers, accounting for 28.06% of the total land area of Jiangxi province, to further maintain the ecological security pattern, clarify the land space control area and promote sustainable development. These practices indicate that Jiangxi province has actively explored various ecological civilization constructions to better promote the integration of green development and rapid development.

The purpose of this study is to examine the ecosystem service values of Jiangxi province from 1995 to 2015 and quantify the trade-off and synergistic relationships among ecosystem services based on the utilization of the ecosystem service trade-off degree. The influencing factors of ecosystem service value changes and the implications of ecosystem sustainability are analyzed and discussed in this paper. Based on the findings of this study, we expect to provide meaningful suggestions for future development in Jiangxi province.

## 2. Study Area and Methods

### 2.1. Study Area

**Figure 1:**
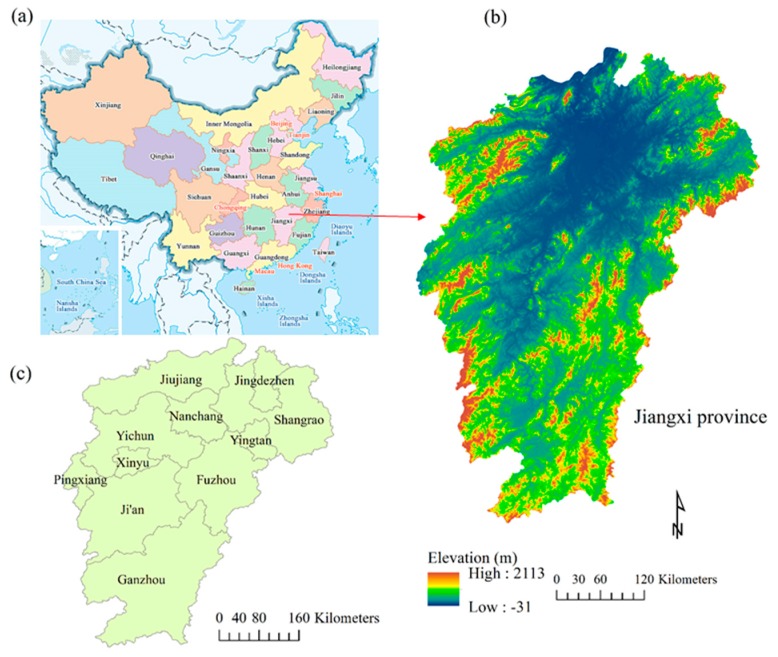
Location of Jiangxi province, China. (**a**) Map of China (source: www.travelchinaguide.com); (**b**) elevation of Jiangxi province (sourced from SRTM images); and (**c**) eleven prefecture-level cities of Jiangxi province.

Jiangxi province (24°29′–30°04′ N, 113°34′–118°28′ E) is located in Central China, and on the southern bank of the junction of the middle and lower reaches of the Yangtze River, covering an area of 16.69 × 10^4^ km^2^, with 11 prefecture-level cities (Figure 1) [26]. Jiangxi is one of the most high ecosystem service value provinces in China [27]. This province is dominated by mountains and hills, accounting for 78% of the total land area of the province [26]. This province has a very high forest coverage rate, which was 63.1% in 2017; and the province is ranked second in forest area behind Fujian province based on statistical data [26].

From 1995 to 2015, the total population of Jiangxi province increased from 40.625 million to 45.656 million; thus, large population growth occurred in the study period, and the urbanization rate (i.e., the proportion of urban population to total population) increased from 23.85% to 51.62% [28]. With the continuous advancement of population urbanization and land urbanization, the ecological environment becomes damaged and ecological functions decline.

Topographically, the terrain inclines from south to north, and from the outskirts to the central part of Poyang Lake (Figure 1). The low elevation area is clearly distributed in central Jiangxi province. There are more than 2400 rivers in the province, with a total length of approximately 18,400 km. Poyang Lake is the largest freshwater lake and one of the top ten ecological function reserves in China. The important ecological protection functions of Jiangxi province were established, and this province was included in the first batch of National Ecological Civilization Pilot Zones in 2016. This province has a subtropical monsoon climate, with a mean temperature of 18.9 °C and an average precipitation of 1739 mm in 2017 [26].

### 2.2. Data

The land cover data for five five-year epochs centered on 1995, 2000, 2005, 2010, and 2015 were delivered from the ESA CCI land cover products. ESA CCI land cover products are based on annual land cover data and provide long-term land cover change information from 1992 to 2015, with a spatial resolution of 300 m [29]. This dataset describes 37 original land cover categories classified based on the United Nations Land Cover Classification System (UN-LCCS) [29,30]. Recently, this dataset has been utilized in many case studies for exploring the availability of land cover information [21,22,23,24]. The object-based validation database of 2600 points or primary sampling units was built to assess the accuracy of land cover categories [29], with an overall weighted-area accuracy of approximately 71.1% [29]. Specifically, the overall accuracy of the ESA CCI land cover data in China was the highest among the coarse resolution datasets [31]. Thus, the utilization of ESA CCI land cover data can provide valuable insights for specific regions of China. In this study, the land cover maps for five years with 20 categories are evaluated, and the land cover classification of Jiangxi province is shown in Table 1.

### 2.3. Landscape Change Index

The landscape change index (LCI) is defined as the absolute values of change in the land cover categories that have the greatest impact on the shape of the landscape, assuming that both the increases and decreases in these values cause changes in the landscape [32,33]. First, the changes in the percentage share of areas covered by each land cover category must be calculated, and the equation is as follows:

(1) $$CA_{i}=100\times \left(A_{t+1}-A_{t}\right)/TA$$

where *CA_i_* represents changes in the percentage share of areas covered by each land cover type in relation to the total land area; *A_t_*_+1_ and *A_t_* represent the area covered with each land cover category during the time interval *t* + 1 and *t*, respectively; and *TA* represents the total land area [32,33].

Then, the landscape change index was calculated (Equation (2)) as follows:

(2) $$LCI_{t}=\frac{1}{2}\times \sum_{i=1}^{n}\left|CA_{i}\right|\;$$

where *LCI_t_* represents the landscape change index in each period; and |*CA_i_*| represents the absolute value of change in the percentage share of the areas covered by each land cover type in relation to the total land area [32,33].

### 2.4. Ecosystem Service Value Estimation

Xie et al. [34] originally proposed the ecosystem services value unit area of Chinese terrestrial ecosystem based on the work by Costanza et al. [1]. It is divided into four primary types (the MEA also classified ecosystem services into four types [4]) and nine secondary types, as shown in the first and second columns of Table 2. Currently, there are many case studies reporting ecosystem service value estimation considering the actual situation of their study area, and they were adopted based on the equivalent value proposed by Xie et al. [34,35], which was carried out through a questionnaire survey among 200 Chinese ecologists. Based on statistical data [26], the average grain production of Jiangxi province from 1995 to 2015 was 5217.37 kg/hm^2^. According to the results of Xie et al. [36], the biomass factor of Jiangxi province was 1.51 [36]. The grain price of Jiangxi province was 2.73 Yuan/kg based on the average value from January to September 2015, which was derived from the Department of Agriculture and Rural Affairs of Jiangxi province. In general, natural food production was proposed to be 1/7 of actual food production [34]. Thus, the economic value of the food production of cropland ecosystems in Jiangxi province was 2034.77 Yuan/hm^2^. Then, the values of each ecosystem service per unit area can be estimated by the product of weight for each ecosystem service and the economic value of the food production of the cropland ecosystem. For more details, the specific values of each ecosystem service per unit area of Jiangxi province are shown in Table 2. A global study of ecosystem service value utilized the ESA CCI land cover products, which classified shrubland into grassland for further estimation [25]; thus, we used the ecosystem service value of shrubland as that of grassland in this paper.

According to the unit price of each ecosystem service function (Table 2) and the area of different land use/cover types, the service value of each ecosystem (Equation (3)), the ecosystem service value of each land use/cover type (Equation (4)), and the total ecosystem service value (Equation (5)) can be calculated based on the following equations.

(3) $$ESV_{i}=\underset{j}{\sum }E_{ij}\times A_{j}\;$$

(4) $$ESV_{j}=\underset{i}{\sum }E_{ij}\times A_{j}\;$$

(5) $$ESV=\underset{i}{\sum }\underset{j}{\sum }E_{ij}\times A_{j}$$

where *A_j_* is the area of land cover type *j*, *ESV_i_* is the economic value of ecosystem service *i* over the study area, *ESV_j_* is the ecosystem service value of land cover type *j*, and *ESV* is the total ecosystem service value of the study area [18,19,20,37].

### 2.5. Coefficient of Sensitivity

To measure the robustness of the estimation results, the coefficient of sensitivity (*CS*) was calculated using the standard economic concept of elasticity [38] to quantitatively describe the sensitivity of the change in ecosystem service value to the value coefficient. Many studies [18,39] have performed a 50% adjustment in the value coefficient. Herein, the ecosystem value coefficient of each land cover type was adjusted by 50% and the change in ecosystem service value was measured. When *CS* > 1, then the *ESV* is elastic to changes in *CS*; when *CS* < 1, then the *ESV* is inelastic to changes in *CS*, which means that the result is credible [18,39,40]. *CS* quantitatively reflects the dependence of output variables on input variables, which verifies the accuracy of the value coefficient we used in the estimation. The calculation of *CS* (Equation (6)) is as follows:

(6) $$CS=\frac{\left(ESV_{j}-ESV_{i}\right)/ESV_{i}}{\left(VC_{jk}-VC_{ik}\right)/VC_{ik}}\;$$

where *CS* is the coefficient of sensitivity; *ESV_i_* and *ESV_j_* represent the initial and adjusted ecosystem service values, respectively; *VC_ik_* and *VC_jk_* represent the initial and adjusted value coefficients, respectively; and *k* is the land cover type [25,40].

### 2.6. Ecosystem Services Trade-Off Degree

The ecosystem services trade-off degree is used to evaluate the interaction of ecosystem service change between two ecosystem services. When the degree is negative, then the relationship between them is a trade-off; when the degree is positive, then the relationship between them is synergistic [41]. The calculation of the ecosystem services trade-off degree (Equation (7)) [15] is as follows:

(7) $$ESTD_{ij}=\frac{ESV_{ib}-ESV_{ia}}{ESV_{jb}-ESV_{ja}}$$

where *ESTD_ij_* is the ecosystem service trade-off degree between ecosystem services *i* and *j*; *ESV_ib_* and *ESV_ia_* are the value of ecosystem services with *i* at time *b* and *a*, respectively; and *ESV_ja_* and *ESV_ja_* are the value of ecosystem services with *j* at time *b* and *a*, respectively. The absolute value of the ecosystem services trade-off degree represents the degree of change in ecosystem services of type *i* compared with that of type *j* [15].

## 3. Results

### 3.1. Land Cover Dynamics of Jiangxi Province

**Figure 2:**
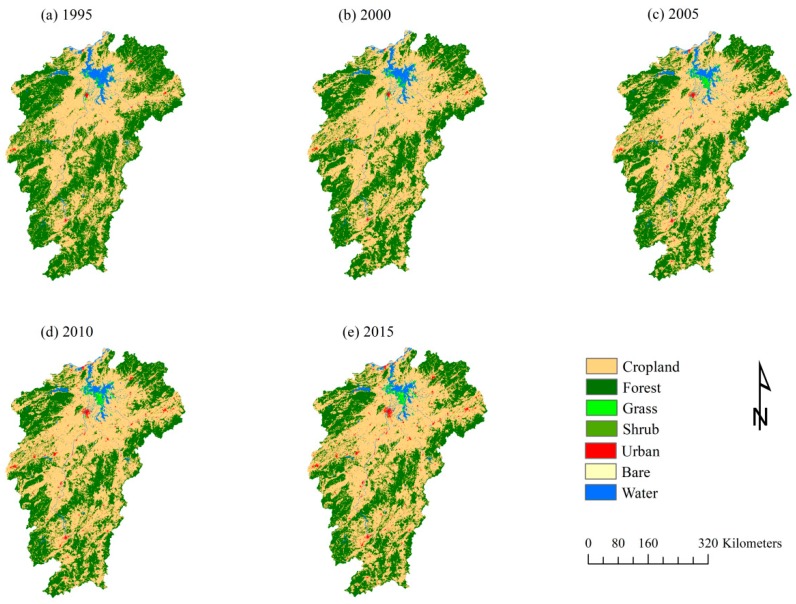
Land cover dynamics of Jiangxi province: (**a**) land cover in 1995; (**b**) land cover in 2000; (**c**) land cover in 2005; (**d**) land cover in 2010; and (**e**) land cover in 2015.

Land cover maps of Jiangxi province are presented in Figure 2, which visually shows that cropland and forest are the dominant land cover types, and shrub and bare land are the minority land cover categories in Jiangxi province. Cropland is distributed in the central area, which is highly associated with low elevation. The forest is located in the northeastern, northwestern, and southern regions of Jiangxi province, which is consistent with the high elevation. The figure clearly shows that the forest land cover has declined during the study periods. The red dots indicating where urban land is located have increased. It is worth mentioning that Nanchang city has greatly increased, which can be clearly observed in 2010 and 2015. Grassland is mainly located in the surrounding area (e.g., lakeshore) of Poyang Lake. The water area is mainly located in Poyang Lake; thus, the shrinkage of Poyang Lake directly affected the decline of the water area in Jiangxi province.

The hectares of land cover in Jiangxi province are statistically shown in Table 3. Cropland increased much more during the 1995–2000 period and tended to be stable with a slight increase from 2000 to 2015. The forest declined from 1995 to 2015, which can be clearly seen in Figure 2. Grassland has expanded and then tended to be stable in the last three study years (i.e., 2005, 2010, and 2015). Urban land drastically increased, especially after 2000, expanding from 846.54 km^2^ in 2000 to 2317.48 km^2^ in 2015, with an average growth rate of 11.58%. Shrubland has greatly declined in the study periods, although it had a turning point in 2000, and appeared to be stable in 2010 and 2015. Bare land slightly increased during the study periods, with 72.21 km^2^ in 2015, which is approximately double the value in 1995. Both shrubland and bare land have a small percentage of the total land area. Water remarkably declined, especially during the 1995–2005 period, which is consistent with the shrinkage of Poyang Lake.

The landscape change index results in Jiangxi province are shown in Table 4. The highest value occurred in the 1995–2000 period, meaning that the landscape transformation intensity was the highest, and the landscape change index value (i.e., 5.16) for this period was more than three times higher than that in the 2000–2005 period. The lowest landscape change index value (i.e., 0.57) was found in Jiangxi province between 2010 and 2015, where the scope of the landscape transformation was the smallest with respect to the total area of Jiangxi. The changes of cropland and forest cover resulted in a high landscape change index value in the 1995–2000 period, and the impact of changes in cropland and forest gradually decreased. Moreover, changes of the percentage share of cropland and urban area gradually decreased and increased, respectively.

### 3.2. Estimation of Ecosystem Service Values

The estimation of ecosystem service values of Jiangxi province is shown in Table 5. The total ecosystem service values decreased from 37.91 × 10^10^ Yuan in 1995 to 35.27 × 10^10^ Yuan in 2015, rapidly declined during the 1995–2000 period and slowed during the 2000–2015 period. The major decline in ecosystem service values resulted from the decrease in forest and water. Cropland ecosystems increased in the study periods, with an increase of 16.80 × 10^9^ Yuan from 1995 to 2015. Slight changes were observed for crop ecosystems between 2010 and 2015. Grassland and bare land ecosystems increased from 1995 to 2010 but slightly declined between 2010 and 2015. The shrubland ecosystem value increased from 1995 to 2000 and suddenly decreased after 2000. The water ecosystem value rapidly declined from 1995 to 2000 and was stable in the latest periods (i.e., 2005–2015).

The values of the ecosystem service functions of Jiangxi province are estimated in Table 6. Regulating services are the primary type of ecosystem services that decreased. Specifically, raw material, gas regulation, climate regulation, water supply, soil formation and retention, biodiversity protection, recreation, and culture were the major secondary types of ecosystem services that declined. It is worth mentioning that water supply had the highest decline in ecosystem service value between 1995 and 2015, with a gap of 6.63 × 10^9^ Yuan. Food production increased in the whole period, especially in the 1995–2000 period. Waste treatment had a fluctuating trend but was more related to a slightly declining trend. In total, these ecosystem services apparently declined between 1995 and 2000 and then tended to be stable or slightly increased or decreased after 2000.

The coefficient of sensitivity of the ecosystem service values of Jiangxi province is shown in Table 7. All values of the coefficient of sensitivity were less than 1, and the highest value was 0.61 (i.e., forest). Therefore, it can be understood that ecosystem service value is inelastic to the coefficient of sensitivity, further indicating that the estimation result is reliable. Forest had the highest value (i.e., 0.61) of the coefficient of sensitivity in 1995 because of the large area of forest and the high coefficient value of ecosystem service per unit area, implying that the total estimated ecosystem service values increased by 0.61% in Jiangxi province when the ecosystem service value coefficient per unit area of forest increased by 1%. The coefficient of sensitivity for forest tended to decrease but was still higher than that for other land cover types. Forest plays the most important role in the total ecosystem service value of Jiangxi province. Cropland was the second most important land cover type for the total ecosystem service value of Jiangxi province. Although water had the highest value of ecosystem services per unit area, the proportion of water area was not very high in these years.

### 3.3. Analysis of the Ecosystem Services Trade-Off Degree

The ecosystem services trade-off degree of Jiangxi province from 1995 to 2015 is shown in Table 8. Based on the result of the whole period, 65 pairs are positive (i.e., synergies) and 16 pairs are negative (i.e., trade-offs). This result indicates that synergy is the primary relationship among ecosystem services in Jiangxi province. Synergy mostly occurs for all ecosystem services except food production, which indicates that the increase of food production would lead to a decrease of other ecosystem functions in Jiangxi province. The trade-off degree between food production and waste treatment was the lowest, and the synergistic degree between water supply and waste treatment was the highest. Therefore, the relationship between food production and waste treatment was most serious among all ecosystem functions.

The result of land cover dynamics showed that there was a drastic change from 1995 to 2000. Thus, we further studied the ecosystem services trade-off degree in the 1995–2000 period and compared it with the direction of interaction of ecosystem services in the 2000–2015 period. The ecosystem services trade-off degree of Jiangxi province between 1995 and 2000 is presented in Table 9. It shows that 53 pairs are positive (i.e., synergies) and 28 pairs are negative (i.e., trade-offs). Synergy is the most common relationship among ecosystem services in Jiangxi province. However, during the 1995–2000 period, trade-offs mainly existed in both food production and waste treatment. The trade-off degree between the water supply and waste treatment was the lowest, and the synergistic degree between food production and waste treatment was the highest. The ecosystem services trade-off degree of Jiangxi province from 2000 to 2015 is shown in Table 10. The results indicate that synergy is the main relationship among ecosystem services in Jiangxi province. Moreover, the relationship between two ecosystem services are consistent with the results of the 1995–2015 period. However, the trade-off degree between climate regulation and food production was the lowest, and the synergistic degree between the water supply and waste treatment was the highest.

## 4. Discussion

### 4.1. Influencing Factors of Ecosystem Service Value Changes in Jiangxi Province

As Table 2 shows, the unit price of forest and water ecosystems is higher than that of the other types, which has greatly impacted the changes in ecosystem service value. Based on the observations, the water area decreased from 1995 to 2015, which clearly demonstrates the shrinkage of Poyang Lake. Thus, the water area declined by 951.4 km^2^, and correspondingly, the ecosystem service value of water declined by 5893.53 × 10^6^ Yuan from 1995 to 2015. As Liu et al. [42] reported, Poyang Lake is undergoing a gradual shrinking trend, which is consistent with the results we obtain in this study. The shrinkage of Poyang Lake was a result of the combined effects of precipitation, evapotranspiration, and outflow discharge [42].

Forest land declined by 12,728.28 km^2^, and correspondingly, the ecosystem service value of forest declined by 37,476.55 × 10^6^ Yuan during the study period. According to the study by Song and Deng [43], the ecosystem service values of forest area significantly declined by 0.45% and 0.10% during the periods of 1988–2000 and 2000–2008, respectively. The declining trend of ecosystem service values in Jiangxi province before 2000 coincided with the trend of the loss of ecosystem service values of China. The subtropical monsoon climate is also an important force, and adequate precipitation mainly occurred in South China, where Jiangxi province is located, and this region features dense vegetation. Furthermore, the most important determinants of forest decreases in Jiangxi province between 1990 and 2010 were soil organic matter, distance to the nearest rural settlement, and slope (<5°) [44].

Urban land also showed an unprecedented expansion from 846.54 km^2^ in 2000 to 2317.48 km^2^ in 2015, with an average growth rate of 11.58% in Jiangxi province. The finding shows that the exploitable land resources (e.g., cropland) were replaced and transferred to urban land. As the landscape change index demonstrates, the change of the percentage share of urban land increased between 1995 and 2015. The over-exploitation of the superior land resources exceeded the ecological carrying capacity, the total ecosystem service values declined and the ecosystem services weakened. Thus, urban development is one of the most important forces affecting the ecosystem services.

It is worth noting that the increase in cropland greatly contributed to the total ecosystem service values of Jiangxi province. The ecosystem service value per unit area for cropland is 14,060.26 Yuan/hm^2^, which is ranked third among all ecosystem types, after water and forest. Thus, both the increase in cropland area and the relatively high economic value for cropland ecosystems relieve the declining gap in the total ecosystem services value.

### 4.2. Ecosystem Sustainability of Jiangxi Province

Jiangxi province is an important ecological security barrier. The forest distribution is well-matched with the high elevation areas. Forest land suddenly decreased between 1995 and 2000 and varied from 78,433.23 km^2^ to 70,126.50 km^2^, which indicates a large decline. Jiangxi province started the Grain for Green Project in 2001, and 11.35 million mu had been completed by the end of 2014, further improving the forest coverage and maintaining land ecological security. This result indicates that the Grain for Green Project has effectively restrained the decrease in forest land in Jiangxi province. Since 2016, Jiangxi province has been listed in the first batch of National Ecological Civilization Pilot Zones, and it can be expected that the natural environment will be improved to a greater extent in the future.

Poyang Lake is the largest freshwater lake in China, one of the top ten ecological function reserves in China, and an important ecological area worldwide. Although the ecological importance of Poyang Lake as a dynamic wetland system is well known [45], the water level and water area in Poyang Lake have declined. Thus, the shrinkage of Poyang Lake has degraded the ecosystem sustainability, resulting in e.g., threatening biological habitats and depressing fisheries resources [46]. The decline of water in Jiangxi province severely threatens agricultural irrigation and then directly influences agricultural production. In addition, because the change of the percentage share of cropland gradually decreased, the potential risk of cropland loss should be monitored in the near future. Agricultural ecosystems are among of the most prominent land types that need to be considered for the implementation of ecological civilization in Jiangxi province.

According to the estimation of ecosystem service functions, raw material, gas regulation, climate regulation, water supply, soil formation and retention, biodiversity protection, and recreation and culture are the major secondary types of ecosystem services that declined from 1995 to 2015. Nevertheless, the loss of the total ecosystem service values was due to the decrease in forest and water areas. Consequently, it is not surprising that the decrease in forest and water areas had a dramatic effect on the estimated total ecosystem service value of Jiangxi province. It is worth mentioning that Jiangxi province formulated an ecological protection red line, which is closely related to water conservation, biodiversity conservation, and soil and water conservation. Thus, the forest and water resources need to be thoroughly considered for the implementation of National Ecological Civilization Pilot Zone in the future. In particular, it is worth noting that raw material, water supply, and recreation and culture are still experiencing relatively high declines of ecosystem service values, warranting the need for future attention.

According to the ecosystem service trade-off degree in Jiangxi province, the proportion of synergy greatly increased in the 2000–2015 period compared with the 1995–2000 period. The synergistic relationship between waste treatment and other ecosystem services increased. However, synergy prominently occurred for all ecosystem services except food production. The trade-off relationship between food production and other ecosystem services has still not improved. The Poyang Lake region also showed that a trade-off relationship occurs between food production and soil conservation and water yield [47]. Thus, we suggest that the relationship between food production and other ecosystem services should be considered in the future.

## 5. Conclusions

This study examined the ecosystem service values of Jiangxi province from 1995 to 2015 based on ESA CCI land cover products. The land cover dynamics have greatly changed under the context of rapid population growth and land urbanization. Forest land declined from 78,433.23 km^2^ in 1995 to 65,704.95 km^2^ in 2015. Correspondingly, urban land drastically increased after 2000, expanding from 846.54 km^2^ in 2000 to 2317.48 km^2^ in 2015. Urban development is one of the most important influencing factors affecting the decline of ecosystem service values in Jiangxi province. The water area remarkably decreased in Jiangxi province, especially from 1995 to 2005, because of the shrinkage of Poyang Lake.

The estimated total ecosystem service values have a decreasing tendency over the whole period. It is worth noting that the value rapidly declined between 1995 and 2000. The ecosystem service values experiencing the greatest declines have resulted from the decreases in forest and water. Regulating services are the dominant type of ecosystem service values that have declined. It is not surprising that food production increased throughout the whole period because of the increase of cropland, especially in the 1995–2000 period, which mitigated the large declining gap in ecosystem service values in the 1995–2000 period.

Synergy was the dominant relationship among ecosystem services in Jiangxi province from 1995 to 2015, and the results indicated that synergy mostly existed in all ecosystem services, except food production. However, during the 1995–2000 period, trade-offs mainly existed in both food production and waste treatment. The proportion of synergy greatly increased in the 2000–2015 period, and the synergistic relationship between waste treatment and other ecosystem services increased. However, the trade-off relationship between food production and other ecosystem services has still not improved significantly. In addition, the change of the percentage share of cropland gradually decreased; thus, the potential risk of cropland loss should be monitored in the future.

It should be stressed that the land cover dynamics were sourced from the ESA CCI land cover products; thus, our analysis results were derived based on this dataset. Findings based on analyses of high-resolution remotely sensed data may provide important insights for exploring ecosystem services at the provincial scale.

## Figures and Tables

**Table 1:** Land cover classification of Jiangxi province.

Land Cover Types	Land Cover Types and Code Numbers Used in the ESA CCI Land Cover Maps	
Cropland	10, 11	Rainfed cropland
	20	Irrigated cropland
	30	Mosaic cropland (>50%)/natural vegetation (tree, shrub, herbaceous cover) (<50%)
	40	Mosaic natural vegetation (tree, shrub, herbaceous cover) (>50%)/cropland (<50%)
Forest	50	Tree cover, broadleaved, evergreen, closed to open (>15%)
	60, 61	Tree cover, broadleaved, deciduous, closed to open (>15%)
	70	Tree cover, needleleaved, evergreen, closed to open (>15%)
	100	Mosaic tree and shrub (>50%)/herbaceous cover (<50%)
	110	Mosaic herbaceous cover (>50%)/tree and shrub (<50%)
	170	Tree cover, flooded, saline water
Grass	130	Grassland
	180	Shrub or herbaceous cover
Shrub	120, 121	Shrubland
Urban	190	Urban areas
Bare	150	Sparse vegetation (tree, shrub, herbaceous cover)
	200	Bare areas
Water	210	Water bodies

Note: adopted from the ESA CCI land cover quick user guide.

**Table 2:** Values of each ecosystem service per unit area in Jiangxi province (unit: Yuan/hm^2^).

Primary Type	Secondary Type	Forest	Grass	Crop	Shrub	Water	Bare
Provision	Food production	134.75	404.26	2034.77	404.26	134.75	13.48
	Raw material	3503.58	67.38	203.48	67.38	13.48	0.00
Regulation	Gas regulation	4716.35	1078.02	1017.39	1078.02	0.00	0.00
	Climate regulation	3638.33	1212.78	1810.95	1212.78	619.86	0.00
	Water supply	4312.10	1078.02	1220.86	1078.02	27,462.66	40.43
	Waste treatment	1765.26	1765.26	3337.02	1765.26	24,498.09	13.48
Support	Soil formation and retention	5255.37	2627.68	2970.76	2627.68	13.48	26.95
	Biodiversity protection	4392.95	1468.81	1444.69	1468.81	3355.35	458.16
Culture	Recreation and culture	1724.84	53.90	20.35	53.90	5848.28	13.48
Total		29,443.53	9756.12	14,060.26	9756.12	61,945.94	565.96

**Table 3:** Hectares of land cover types in Jiangxi province from 1995 to 2015 (unit: km^2^).

	1995	2000	2005	2010	2015
Crop	81,030.64	89,217.22	91,065.89	92,538.06	92,980.14
Forest	78,433.23	70,126.50	68,527.30	66,653.82	65,704.95
Grass	969.22	1113.38	1430.16	1495.65	1486.83
Shrub	474.13	646.76	143.11	76.40	72.74
Urban	739.23	846.54	1305.74	1808.68	2317.48
Bare	36.50	45.14	67.06	73.17	72.21
Water	5305.20	4992.61	4448.89	4342.36	4353.80

**Table 4:** Landscape change index (LCI) in Jiangxi province from 1995 to 2015.

Time Interval	Indicator	Crop	Forest	Grass	Shrub	Urban	Bare	Water
1995–2000	CA (%)	4.90	−4.97	0.09	0.10	0.06	0.01	−0.19
	LCI	5.16						
2000–2005	CA (%)	1.11	−0.96	0.19	−0.30	0.27	0.01	−0.33
	LCI	1.58						
2005–2010	CA (%)	0.88	−1.12	0.04	−0.04	0.30	0.00	−0.06
	LCI	1.23						
2010–2015	CA (%)	0.26	−0.57	−0.01	0.00	0.30	0.00	0.01
	LCI	0.57						

Note: CA: the changes in the percentage share of areas covered by each land cover category; LCI: landscape change index.

**Table 5:** Ecosystem service values of Jiangxi province from 1995 to 2015 (unit: 10^6^ Yuan).

	1995	2000	2005	2010	2015
Crop	113,931.19	125,441.74	128,041.02	130,110.92	130,732.50
Forest	230,935.09	206,477.14	201,768.54	196,252.35	193,458.54
Grass	945.58	1086.23	1395.28	1459.17	1450.57
Shrub	462.57	630.99	139.62	74.54	70.97
Bare	2.07	2.55	3.80	4.14	4.09
Water	32,863.56	30,927.19	27,559.07	26,899.16	26,970.03
Total	379,140.06	364,565.84	358,907.32	354,800.28	352,686.69

**Table 6:** Values of ecosystem service functions of Jiangxi province from 1995 to 2015 (unit: 10^6^ Yuan).

Primary Type	Secondary Type	1995	2000	2005	2010	2015
Provision	Food production	17,674.67	19,237.12	19,576.88	19,849.71	19,926.53
	Raw material	29,145.35	26,403.31	25,878.65	25,252.06	24,928.55
Regulation	Gas regulation	45,391.42	42,340.72	41,754.41	41,020.46	40,616.57
	Climate regulation	43,714.70	42,194.03	41,890.60	41,468.82	41,202.84
	Water supply	58,439.11	55,032.37	53,055.22	52,134.42	51,809.30
	Waste treatment	54,137.21	54,692.88	53,662.52	53,561.88	53,567.73
Support	Soil formation and retention	65,678.34	63,827.73	63,486.72	62,939.03	62,568.43
	Biodiversity protection	48,155.44	45,631.09	44,986.76	44,340.79	43,989.79
Culture	Recreation and culture	16,803.80	15,206.59	14,615.55	14,233.10	14,076.96
Total		379,140.06	364,565.84	358,907.32	354,800.28	352,686.69

**Table 7:** Coefficient of sensitivity of the ecosystem service values of Jiangxi province.

	1995		2000		2005		2010		2015	
	%	CS	%	CS	%	CS	%	CS	%	CS
Crop *VC*±50%	15.02	0.30	17.20	0.34	17.84	0.36	18.34	0.37	18.53	0.37
Forest *VC*±50%	30.46	0.61	28.32	0.57	28.11	0.56	27.66	0.55	27.43	0.55
Grass *VC*±50%	0.12	0.00	0.15	0.00	0.19	0.00	0.21	0.00	0.21	0.00
Shrub *VC*±50%	0.06	0.00	0.09	0.00	0.02	0.00	0.01	0.00	0.01	0.00
Bare *VC*±50%	0.00	0.00	0.00	0.00	0.00	0.00	0.00	0.00	0.00	0.00
Water *VC*±50%	4.33	0.09	4.24	0.08	3.84	0.08	3.79	0.08	3.82	0.08

Note: 0.00 indicates that the value is quite small and very close to zero but not zero. CS: coefficient of sensitivity.

**Table 8:** Ecosystem services trade-off degrees in Jiangxi province from 1995 to 2015.

	FP	RM	GR	CR	WS	WT	SFR	BP	RC
FP	1.00	−0.53	−0.47	−0.90	−0.34	−3.95	−0.72	−0.54	−0.83
RM	−0.53	1.00	0.88	1.68	0.64	7.40	1.36	1.01	1.55
GR	−0.47	0.88	1.00	1.90	0.72	8.38	1.54	1.15	1.75
CR	−0.90	1.68	1.90	1.00	0.38	4.41	0.81	0.60	0.92
WS	−0.34	0.64	0.72	0.38	1.00	11.64	2.13	1.59	2.43
WT	−3.95	7.40	8.38	4.41	11.64	1.00	0.18	0.14	0.21
SFR	−0.72	1.36	1.54	0.81	2.13	0.18	1.00	0.75	1.14
BP	−0.54	1.01	1.15	0.60	1.59	0.14	0.75	1.00	1.53
RC	−0.83	1.55	1.75	0.92	2.43	0.21	1.14	1.53	1.00

Note: FP: food production; RM: raw material; GR: gas regulation; CR: climate regulation; WS: water supply; WT: waste treatment; SFR: soil formation and retention; BP: biodiversity protection; RC: recreation and culture.

**Table 9:** Ecosystem services trade-off degrees in Jiangxi province from 1995 to 2000.

	FP	RM	GR	CR	WS	WT	SFR	BP	RC
FP	1.00	−0.57	−0.51	−1.03	−0.46	2.81	−0.84	−0.62	−0.98
RM	−0.57	1.00	0.90	1.80	0.80	−4.93	1.48	1.09	1.72
GR	−0.51	0.90	1.00	2.01	0.90	−5.49	1.65	1.21	1.91
CR	−1.03	1.80	2.01	1.00	0.45	−2.74	0.82	0.60	0.95
WS	−0.46	0.80	0.90	0.45	1.00	−6.13	1.84	1.35	2.13
WT	2.81	−4.93	−5.49	−2.74	−6.13	1.00	−0.30	−0.22	−0.35
SFR	−0.84	1.48	1.65	0.82	1.84	−0.30	1.00	0.73	1.16
BP	−0.62	1.09	1.21	0.60	1.35	−0.22	0.73	1.00	1.58
RC	−0.98	1.72	1.91	0.95	2.13	−0.35	1.16	1.58	1.00

Note: FP: food production; RM: raw material; GR: gas regulation; CR: climate regulation; WS: water supply; WT: waste treatment; SFR: soil formation and retention; BP: biodiversity protection; RC: recreation and culture.

**Table 10:** Ecosystem services trade-off degrees in Jiangxi province from 2000 to 2015.

	FP	RM	GR	CR	WS	WT	SFR	BP	RC
FP	1.00	−0.47	−0.40	−0.70	−0.21	−0.61	−0.55	−0.42	−0.61
RM	−0.47	1.00	0.86	1.49	0.46	1.31	1.17	0.90	1.31
GR	−0.40	0.86	1.00	1.74	0.53	1.53	1.37	1.05	1.53
CR	−0.70	1.49	1.74	1.00	0.31	0.88	0.79	0.60	0.88
WS	−0.21	0.46	0.53	0.31	1.00	2.86	2.56	1.96	2.85
WT	−0.61	1.31	1.53	0.88	2.86	1.00	0.89	0.69	1.00
SFR	−0.55	1.17	1.37	0.79	2.56	0.89	1.00	0.77	1.11
BP	−0.42	0.90	1.05	0.60	1.96	0.69	0.77	1.00	1.45
RC	−0.61	1.31	1.53	0.88	2.85	1.00	1.11	1.45	1.00

Note: FP: food production; RM: raw material; GR: gas regulation; CR: climate regulation; WS: water supply; WT: waste treatment; SFR: soil formation and retention; BP: biodiversity protection; RC: recreation and culture.
